# Genome-wide identification of ethylene receptor protein-coding gene families in wheat and their regulated expression during development and under multiple abiotic stresses

**DOI:** 10.1186/s12870-026-08177-7

**Published:** 2026-01-26

**Authors:** Murali Krishna Koramutla, Manisha Negi, Deepak Sharma, Belay T. Ayele

**Affiliations:** 1 Department of Plant Science, University of Manitoba, 222 Agriculture Building, Winnipeg, MB R3T 2N2 Canada; 2 Current Address: Agriculture and Agri-Food Canada, Lethbridge Research and Development Centre, Lethbridge, AB T1J 4B1 Canada; 3 Current address: Corteva Agriscience, Lethbridge, AB T1J 5N9 Canada

**Keywords:** Ethylene receptor proteins, Gene expression, Gene family, Gene structure, Abiotic stress, Wheat

## Abstract

**Background:**

Ethylene regulates many plant growth and developmental processes and mediates plant-environment interactions. Ethylene signal in plants is perceived by transmembrane ethylene receptor proteins (ETRs), which initiate a cascade downstream signaling events that regulate the expression of ethylene responsive genes. However, the identity and roles of ETRs in wheat remain to be investigated. This study performed genome-wide identification and characterization of *ETR* genes in hexaploid wheat (*Triticum aestivum* L.) and its tetraploid and diploid progenitors.

**Results:**

A total of 18 homoeologues of six *TaETR* genes were identified in the genome of hexaploid wheat, namely *TaERS1*, *TaERS2*, *TaETR2*, *TaETR3*, *TaETR4* and *TaETR5*, which are distributed unevenly over 4 chromosomes in each of the three subgenomes. These genes typically consist of 1 to 7 exons and 0 to 6 introns, and the exons/introns number in *TaETR* homoeologues varies with their subgenome origin. The TaETR proteins consist of a minimum of 5 and a maximum of 7 domains, of which cGMP phosphodiesterases/adenylyl cyclases/FhlA (GAF) is a conserved domain. Analysis of the promoter sequences of *TaETR* genes revealed the presence of eight groups of cis-regulatory elements, which includes hormone and stress responsive elements. Using publicly available transcriptomic data and qRT-PCR, we found that *TaETR* genes exhibit tissue and stage specific expression patterns during wheat development and differential response to multiple abiotic stresses. The study also characterized *ETR* genes from the tetraploid and diploid progenitors of hexaploid wheat, *T. turgidum* ssp. dicoccoides and *Aegilops tauschii*, respectively.

**Conclusion:**

Ethylene is an important regulator of crop traits of agronomic and economic importance. This study identified *ETR* genes of wheat and predicted their potential roles during wheat growth and development as well as its interaction with the surrounding environment.

**Supplementary Information:**

The online version contains supplementary material available at 10.1186/s12870-026-08177-7.

## Background

Wheat is an important cereal crop that serves as a major staple food for ~ 40% of the global population [1]. However, its production is severely affected by a range of abiotic stress factors including drought, heat, cold, salinity and waterlogging/flooding. Drought and salinity are reported to cause 32% and 50% global wheat yield loss, respectively [2, 3]. Wheat is also highly sensitive to heat stress, and prediction models estimate a 6% reduction in global wheat production for each 1 °C rise in temperature [4]. Low temperature frosts are also reported to affect crop growth and thereby cause significant reduction in wheat yield [5]. It has been reported that flooding/waterlogging affects 10–15 million ha of wheat growing areas every year globally [6], causing 50–86% yield loss depending on crop developmental stage, genotype, soil type, and duration of flooding/waterlogging [7–9].

Abiotic stresses adversely affect plant growth and development, eventually compromising crop yield and quality. Plants respond to abiotic stresses through various morpho-physiological, biochemical and anatomical adjustments [10–13]. For example, switching on energy conserving metabolic pathways and morpho-anatomical adjustments such as formation of adventitious roots and aerenchyma take place in response to waterlogging conditions [13]. Plant response to drought has been reported to involve osmotic adjustment and changes in root morphology [11] while heat stress induces changes in leaf orientation, membrane lipid composition, transpiration-induced cooling and increases in stomata and trichome densities [10]. Under salinity stress, plants mainly adjust cell osmatic potential and ion homeostasis [12].

Ethylene regulates a wide range of plant growth and developmental processes and mediates plant response to a range of biotic and abiotic stress factors [14–16]. Previous studies showed that exogenous ethylene enhances chlorophyll content, fresh weight and antioxidant responses of plants such as rice under heat stress [17, 18]. Similarly, it increases the survival rate of plants such as wheat under drought stress through improved shoot biomass and relative water content [19] and enhances seedling growth under salinity stress via increasing chlorophyll content and reducing oxidative stress [20]. Ethylene also increases chilling/cold tolerance in different crop species such as grapevine or wheat, for example, through increasing antioxidant enzyme activity [21, 22]. Furthermore, it enhances aerenchyma formation, adventitious root development, hyponasty and chlorophyll content in response to waterlogging in several plant species including wheat [23–25].

Genetic studies have also demonstrated the importance of ethylene in triggering stress induced adaptive responses. For example, ethylene insensitive mutant of tomato, *Never ripe* (*Nr*), exhibits fewer adventitious roots when exposed to flooded/waterlogged conditions [23] and hypersensitivity to heat and salinity stress [26, 27]. Likewise, ethylene deficient mutants, *aco1* and *aco3*, of petunia exhibit enhanced sensitivity to drought and salinity [28]. The expression of *alcohol dehydrogenase* gene, which encodes an enzyme involved in anaerobic respiration and is upregulated by waterlogging-induced hypoxia but repressed in ethylene insensitive *etr1-1* and *ein2-1* mutants of Arabidopsis [29], and these two mutants exhibit reduced survival rate under heat stress [30]. Ethylene also acts as a positive or negative regulator of plant response to cold stress. For example, ethylene insensitive mutant *etr2b* of zucchini exhibit enhanced chilling tolerance [31] and ethylene-insensitive receptor mutants of Arabidopsis, *etr1-1* and *ein4-1*, show increased tolerance to freezing stress under clod acclimated or non-acclimated conditions [32]. On the other hand, overexpression of *ETR4* of rice leads to significantly increased survival rate of seedlings in response to cold treatment [33]. Furthermore, cold-tolerant rice varieties exhibit higher expression levels of *ETR* genes than the corresponding cold sensitive varieties [34, 35].

Ethylene signal in plants is perceived by transmembrane receptors localized in the endoplasmic reticulum, golgi apparatus and plasma membranes [36–38]. The model plant Arabidopsis consist of five ethylene receptors (ETRs), namely ethylene response 1 (ETR1), ETR2, ethylene response sensor 1 (ERS1), ERS2, and ethylene insensitive (EIN4) [39]. In the absence of ethylene, ETRs activate constitutive triple response 1 (CTR1) protein, a Raf-like kinase that supress the down stream signalling cascade [38]. Upon their binding with ethylene, these receptors are inactivated, which in turn inhibits EIN2 phosphorylation via CTR1 protein and thereby induces proteolytic cleavage of EIN2 at C-terminus [40]. The cleaved EIN2 C-terminal end moves to the nucleus and induces the degradation of F-box protein EBF1/2, leading to stabilization of the EIN3/EIL1 transcription factors, which activate ethylene response factor 1 (ERF1) that targets ethylene responsive genes. Based on their structural features, ETRs are generally classified into two subfamilies, subfamily I and II. ETRs in both subfamilies consist of three transmembrane domains, a cGMP phosphodiesterases/adenylyl cyclases/FhlA (GAF) domain and a His kinase (HisKA) domain. ETRs of subfamily II possess additional features/domains, which include an N-terminal putative signal peptide, diverged Histidine kinase-, DNA gyrase B-, and HSP90-like ATPase (HATPase_c) domain and a receiver domain. Notably, ETR1-type receptors are not present in monocot species. For example, the five ETR members of rice include ERS1, ERS2, ETR2, ETR3 and ETR4, which are divided into two subfamilies based on their conserved domains as subfamily I (ERS1 and ERS2) and subfamily II (ETR2, ETR3 and ETR4) [41]. Recent studies reported that the GAF domain is conserved in both subfamilies of ETR in many plant species including Arabidopsis [39], rice [41], tomato [42] and soybean [43]. However, ETRs of the two subfamilies do not have equal functional contribution in ethylene signalling. For example, loss of function mutation in *ETR*s of subfamily I results in a much stronger ethylene response phenotype compared to that caused by loss of function mutation in ETRs of subfamily II [44]. Furthermore, complementation of null mutants of subfamily I *ETR*s with subfamily II *ETR*s can not rescue the severe phenotype of the null mutant, indicating subfamily I ETRs perform a core function in ethylene signalling [44].

ETRs are encoded by a multigene family in many plant species including Arabidopsis [39], rice [41], tomato [42] and soybean [43]. However, the *ETR* genes of wheat remain to be identified and as such their structural characteristics and evolutionary relationships are unknown. Hexaploid wheat (*Triticum aestivum*; AABBDD genome) has a complex polyploidy genome, which evolved through hybridization between tetraploid wheat (*T. turgidum*, ssp. *dicoccum*; AABB genome) and diploid wheat (*Aegilops tauschii*; DD genome) [45]. Homoeologous genes of polyploid genomes, which are inherited from distinct progenitors through interspecific hybridization [46], have been reported to exhibit tissue and stage specific expression patterns and distinct modulation of their expression in response to biotic and abiotic stress factors [47–51]. Furthermore, homoeologue expression bias and dominance are reported as common phenomena in polyploids such as cotton, wheat and mustard [47, 49, 52, 53], indicating the importance of specific homoeologues in controlling important traits in such plant species.

Using in silico analysis of publicly available wheat genome sequence information, the present study identified *ETR* genes of hexaploid wheat and its tetraploid and diploid progenitors, *T. turgidum* and *Ae. tauschii*, respectively, and investigated their chromosomal distribution, structure, duplication events and phylogenetic relationships. The study also examined physical and chemical properties as well as conserved domains of the corresponding proteins to better understand their structural features and evolutionary relationships. To predict their potential roles during wheat plant development and its response to abiotic stress, we analyzed the expression patterns of hexaploid wheat *ETR*s in different tissues and developmental stages, and in response to multiple abiotic stress factors.

## Methods

### Genome-wide identification of ethylene receptors genes in wheat

ETRs proteins of hexaploid wheat (TaETRs) were identified using HMMER software (https://www.ebi.ac.uk/Tools/hmmer/) through domain-based screening. The Hidden Markov model (HMM) profile of the GAF domain (Pfam ID: PF01590) was downloaded from the Ensembl Plants database (https://plants.ensembl.org/index.html) and used as a query to search for potential TaETR candidates against the wheat proteome sequence database (https://plants.ensembl.org/Triticum_aestivum/Info/Index) using HMMER. The resulting protein sequences were further screened for the presence of ETR characteristic domains including HisKA (phospho-acceptor) domain (Pfam ID: PF00512) and Histidine kinase-, DNA gyrase B-, and HSP90-like ATPase (HATPase_c) domain (Pfam ID: PF02518) using Simple Modular Architecture Research Tool (SMART) [54]. Proteins lacking one or two domains other than GAF but exhibited high sequence similarity to known ETRs of other plant species such as Arabidopsis and rice were also considered in our analysis not to miss potentially truncated or divergent yet functionally relevant ETRs. The genomic DNA, cDNA and coding sequences (CDS) of the putative *TaETR*s as well as the amino acid sequences of candidate TaETRs were obtained from Ensembl Plants database. Molecular weights (MW) and isoelectric points (*p*I) of the proteins were calculated using the ExPASy online tool (http://web.expasy.org/compute_pi/). Subcellular localizations of the ETR proteins were predicted using CELLO v2.5 [55].

### Analysis of gene structure, cis-regulatory elements, conserved domains and phylogeny

Exon–intron structures of the *ETR* genes of hexaploid wheat and its progenitors were constructed by aligning the CDS and corresponding genomic DNA sequences using the Gene Structure Display Server 2.0 (GSDS) [56]. Conserved domains of the TaETR protein sequences were identified using SMART [54] and their transmembrane architecture was predicted using TMHHM 2.0 (http://www.cbs.dtu.dk/services/TMHMM/). Amino acid sequences of ETRs from other plant species were also retrieved from NCBI GenBank database using their accession numbers, and alignment of the sequences was carried out using Clustal X2 [57]. Phylogenetic tree was constructed using the Neighbor-Joining methods in Molecular Evolutionary Genetics Analysis (MEGA; v10.1.8) with 1000 bootstrap replication [58]. The TaETRs were classified into two subfamilies based on the presence or absence of conserved functional domains in comparison to ETRs of Arabidopsis and rice as well as their phylogenetic relationships with the Arabidopsis and rice ETRs. Cis-regulatory elements were predicted in the 1.5 kb region upstream of the start codon ATG of each *TaETR* gene using PlantCARE database [59].

### Chromosomal distribution, gene duplication events and synteny analysis

Chromosomal location of the *TaETR*s was obtained from the Generic Feature Format Version 3 (GFF3) file in the International Wheat Genome Sequencing Consortium (IWGSC) RefSeq v1.0 database and then the genes were mapped on to their respective chromosomes using MapChart 2.3 [60]. Gene duplications events were identified using the criteria described in Kong et al. [61]. Accordingly, gene sequences with > 80% coverage of the aligned region, > 80% sequence identity, E-value threshold ≤ 10^−10^, and occurrence of only one duplication for closely linked genes on the same chromosome (intergenic distance < 25 kb) were considered as tandem duplications. In addition, gene pairs that satisfy the first three criteria but located on different chromosomes were considered as segmental duplications. To estimate duplication events, the non-synonymous (Ka), synonymous (Ks) and Ka/Ks ratio of duplicated gene pairs were calculated using Simple Ka/Ks Calculator available at TBtools platform [62]. Furthermore, divergence time (T) in millions of years (Mya) was determined using the following formula.

$$T=Ks/2\lambda \times {10}^{-6}Mya$$

where λ = 6.5 × 10^−9^ and it represents the rate of replacement of synonymous substitutions per site per year in plants [63].

TBtools inbuilt multiple collinear scanning tool kit (MCScanX) was used to analyze gene duplication events and synteny relationships among hexaploid wheat (*T. aestivum*), Tausch's goatgrass (*Ae. tauschii*), barley (*Hordeum vulgare*), maize (*Zea mays*), rice (*Oryza sativa*) and tomato (*Solanum lycopersicum*).

### Analysis of expression patterns of TaETR genes in different tissues at developmental stages

The annotated and mapped *TaETR* sequences were compared to obtain the latest version of gene models available at expVIP platform (https://www.wheat-expression.com/). Expression data, in transcripts per million (TPM) values, of the *TaETR* genes in different tissues and at different developmental stages, namely roots at Zadoks [Z] stages Z10, Z13 and Z39; leaves at Z10, Z23 and Z71; stems at Z30, Z32 and Z65; spikes at Z32, Z39 and Z65, and grains at Z71, Z75 and Z85 were extracted from publicly available RNA-seq data using expVIP platform [64]. The extracted TPM values were log2 transformed and converted to expression values in log_2_ fold change for constructing a heatmap and hierarchical clustering using the MultiExperiment Viewer (MeV) software v4.9.0.

### Plant materials, and abiotic stress and ethylene treatment assays

#### Salt and drought stress, and ethylene treatment

Mature dry seeds of wheat cv. Harvest were surface sterilized and imbibed in Petri-plates for three days. Seedlings were then transferred and grown in a hydroponic system containing half-strength Hogland’s nutrient solution described previously [65] at 22 °C/20 °C under 16 h light/8 h dark cycle. The nutrient solution was aerated continuously using an aquarium pump. Seven days after their transfer to a hydroponic system, seedlings were subjected to salt or drought stress by transferring them to hydroponic solution containing either 200 mM NaCl or 20% (w/v) of polyethylene glycol (PEG8000), respectively, while control plants were transferred to a fresh hydroponic solution. Control seedlings and those subjected to salt or drought stress were treated (in the form of spray) with or without ethephon (C2H6ClO3P; Sigma-Aldrich, St. Louis, MO, USA), an ethylene releasing compound, 24 h before they are subjected to the stress treatments. The ethephon solution (100 µM) was prepared using 0.1% (v/v) Tween-20, thus, the control seedlings without ethephon treatment were sprayed with 0.1% (v/v) Tween-20 solution. Whole seedlings were harvested in liquid N_2_ at 0, 1 and 3 days after drought or salt treatment and stored at −80 °C until further use. Shoot and root morphological traits of seedlings including shoot and root length, fresh weight and dry weight as well as leaf chlorophyll content were recorded after 3 days of stress treatment. Leaf chlorophyll content was determined using a SPAD Plus Chlorophyll Meter (Konica Minolta Inc, Japan).

#### Heat stress and cold acclimation, and ethylene treatment

Mature seeds of wheat cv. Harvest were surface sterilized and cultured in Petri-plates as described previously [66] at 22 °C under 16 h light/8 h dark cycle. Seven days old seedlings were subjected to cold (4 °C) or heat (42 °C) stress while the corresponding control seedlings were kept at 22 °C. Control seedlings and seedlings exposed to cold or heat were treated with or without ethephon (100 µM; Sigma-Aldrich) solution 24 h prior to the application of cold or heat treatments. Control seedlings with no ethephon treatment were treated 0.1% (v/v) Tween-20 solution. Shoot and root morphological traits of seedlings including shoot and root length, fresh weight and dry weight as well as leaf chlorophyll content were recorded after 3 days of cold or heat treatment. Leaf chlorophyll content was determined using a SPAD-502 Plus Chlorophyll Meter (Konica Minolta). Whole seedling samples were harvested in liquid N_2_ at 0, 4 and 24 h after the cold or heat treatment and stored at −80 °C until needed for further use.

#### Waterlogging stress

Mature dry seeds of wheat cv. Harvest were first surface sterilized and imbibed for three days, and then seedlings were transferred to three-liter plastic pots containing mixture of soil and sand (2:1) with 18 g of fertilizer (ACER®nt 13–12-12 consisting of 13% N, 12% P2O5, 12% K2O and micro elements) and grown at 22 °C/20 °C under 16 h light/8 h dark cycle as described previously [67]. Thirty-day-old plants were subjected to waterlogging treatment as described previously [68]. After 7 and 14 days of waterlogging, the root and stem node tissues were harvested in liquid N_2_ and stored at −80 °C until further use.

#### RNA isolation, cDNA synthesis and qRT-PCR analysis

Total RNA was extracted using TRIzol reagent (Thermo Fisher Scientific, Waltham, MA, USA) from seedlings exposed to salinity, drought, cold and heat stresses, as well as from root and stem node tissues of waterlogged plants and their respective controls following the manufacturer’s protocol. DNase treatment, cDNA synthesis and qPCR assays were performed as described before [67]. qPCR assays were conducted on CFX96 Real-Time PCR system (Bio-Rad, Hercules, CA, USA) using reaction mix consisting of 5 µl of diluted cDNA (1:20 v/v), 10 μL of 2 × EvaGreen Supermix (Bio-Rad), and 0.6 µl of each forward and reverse primer (10 µM each; 300 nM final concentration for each). The final reaction volume was adjusted to 20 µl with nuclease-free water. The thermal cycling conditions of the qPCR assay involved initial denaturation at 95 °C for 30 s, followed by 40 cycles of denaturation at 95 °C for 5 s, annealing at 60 °C for 30 s and extension at 72 °C for 30 s. Primer sequences of each *TaETR* gene (Table S1) were designed from a region conserved across its three homoeologues using a primer-BLAST tool (https://www.ncbi.nlm.nih.gov/tools/primer-blast/index.cgi), and gene specificity of the primer sequences was verified through RT-PCR (See supplementary Figure S1 for review purpose only). The *Ta18S rRNA* gene (GenBank ID: D86933) was used as reference gene for normalization, and the relative gene transcript levels were determined using the method described previously [69].

### Statistical analysis

Statistically significant differences among samples were determined using two-way ANOVA and Fisher’s least significant difference (LSD) test at *P* < 0.05.

## Results

### ETR gene family members of hexaploid wheat and their chromosomal location

Blast searching of the hexaploid wheat genome sequence database against the amino acid sequence of the GAF domain led to the identification of 167 protein sequences, of which 78 protein sequences were found to consist of the GAF domain and at least one more of the characteristic domains of ETR proteins. These candidate protein sequences were further examined for the presence of the characteristic domain GAF and other domains of the ETRs using SMART and verified for their similarity with previously identified ETRs of Arabidopsis and rice. Genes encoding the protein sequences consisting of ETR characteristic domains and exhibiting high sequence similarity with ETR protein sequences of Arabidopsis and rice were considered as putative *TaETR* genes. Consequently, 18 ETR family members containing ETR characteristic domains were extracted for further study, and these 18 genes were grouped into six putative *TaETR* gene families, each family consisting of a homoeologue derived from each of the three subgenomes of hexaploid wheat (Table 1). The gene families were named after their corresponding homologs in rice. We also identified six *ETR* genes from *T. turgidum* (*TtETR*), each having homoeologues in each of the two subgenomes, and six *ETR* genes from *Ae. tauschii* (*AeETR*) (Table 1). Our analysis showed that the hexaploid wheat *ETR* genes have their respective homoeologues originated from each of its progenitors, AA genome derived from *T. urartu* and the BB genome from an unknown close relative of the diploid *Ae. Speltoides* [70], which together form the tetraploid *T. turgidum* [(AABB), and DD genome derived from *Ae. tauschii*.

**Table 1:** Characteristic features of *ETR* gene family members in common wheat and its progenitor species

Species	Gene name	Gene ID	Gene length (bp)	CDS length (bp)	Protein size (aa)	pI	MW (kDa)	Subcellular localization
*T. aestivum*	*TaERS1A*	TraesCS4A02G274300	2493	1908	635	6.48	70.98	PM*
	*TaERS1B*	TraesCS4B02G039300	2850	1908	635	6.36	71.00	PM
	*TaERS1D*	TraesCS4D02G036400	2665	1902	633	6.48	71.01	PM
	*TaERS2A*	TraesCS1A02G096600	2861	1917	638	7.66	70.20	PM
	*TaERS2B*	TraesCS1B02G127000	2952	1917	638	7.07	70.16	PM
	*TaERS2D*	TraesCS1D02G105600	2748	1920	639	7.07	70.30	PM
	*TaETR2A*	TraesCS2A02G000200	3295	2337	778	6.22	86.70	PM
	*TaETR2B*	TraesCS2B02G023800	3462	2310	769	6.18	85.69	PM
	*TaETR2D*	TraesCS2D02G000500	2810	2331	776	6.22	86.60	PM
	*TaETR3A*	TraesCS6A02G399400	3655	2430	809	7.89	89.31	PM
	*TaETR3B*	TraesCS6B02G439600	3793	2355	784	7.25	86.45	PM
	*TaETR3D*	TraesCS6D02G383600	3505	2355	784	6.98	86.31	PM
	*TaETR4A*	TraesCS6A02G329400	2557	2193	730	8.07	78.35	PM
	*TaETR4B*	TraesCS6B02G360200	2639	2187	728	8.08	78.23	PM
	*TaETR4D*	TraesCS6D02G308300	2187	2187	728	8.39	78.40	PM
	*TaETR5A*	TraesCS6A02G285800	2661	2187	728	8.44	78.05	PM
	*TaETR5B*	TraesCS6B02G314600	2596	2190	729	7.91	78.15	PM
	*TaETR5D*	TraesCS6D02G266200	2550	2181	726	8.44	77.63	PM
*T. turgidum*	*TtERS1A*	TRITD4Av1G194970	1908	1908	635	6.48	70.99	PM
	*TtERS1B*	TRITD4Bv1G011070	1683	1683	560	6.42	62.82	PM
	*TtERS2A*	TRITD1Av1G039460	1917	1917	638	7.66	70.17	PM
	*TtERS2B*	TRITD1Bv1G052020	1917	1917	638	7.07	70.17	PM
	*TtETR2A*	TRITD2Av1G000170	2373	2373	778	6.22	86.70	PM
	*TtETR2B*	TRITD2Bv1G001000	1893	1893	630	6.28	70.72	PM
	*TtETR3A*	TRITD6Av1G220960	2430	2430	809	8.29	89.25	PM
	*TtETR3B*	TRITD6Bv1G221990	2355	2355	784	6.98	86.57	PM
	*TtETR4A*	TRITD6Av1G198620	2193	2193	730	8.07	78.35	PM
	*TtETR4B*	TRITD6Bv1G193080	2187	2187	728	8.08	78.25	PM
	*TtETR5A*	TRITD6Av1G179750	2187	2187	728	8.44	78.05	PM
	*TtETR5B*	TRITD6Bv1G168450	2184	2184	727	8.42	78.06	PM
*Ae. tauschii*	*AetERS1D*	AET5Gv20005900	2685	1902	633	7.34	70.15	PM
	*AetERS2D*	AET1Gv20252500	2876	1920	639	7.07	70.30	PM
	*AetETR2D*	AET0Gv20006200	2772	2334	777	6.22	86.72	PM
	*AetETR3D*	AET6Gv20968100	3723	2335	784	7.61	86.48	PM
	*AetETR4D*	AET6Gv20812200	2384	2088	845	8.92	90.65	PM
	*AetETR5D*	AET6Gv20720200	2530	2181	726	8.44	77.64	PM

^*^plasma membrane

Comparison of the nucleotide sequences of the *TaETR* genes with those of its progenitors revealed that the putative *TaETR* genes consist of 2187 to 3793 bp, while those of *TtETR*s and *AetETR*s genes consist of 1683 to 2430 bp and 2384 to 3723 bp, respectively. The proteins encoded by the putative *TaETR* genes contain 633 to 809 amino acids (aa) with molecular mass ranging from 70.98 to 89.31 kDa and isoelectric points (pI) ranging from 6.48 to 8.44 (Table 1). The TtETR proteins consist of 560 to 809 aa with molecular mass ranging from 62.82 to 89.25 kDa and pIs from 6.42 to 8.29 while the AetETRs consist of 633 to 845 aa with molecular mass ranging from 70.15 to 90.65 kDa and pIs from 7.34 to 8.92. Prediction of the subcellular localization of the ETR proteins revealed that they are all located on the plasma membrane (Table 1).

### Chromosomal distribution and genomic duplication of TaETR genes

**Fig. 1:**
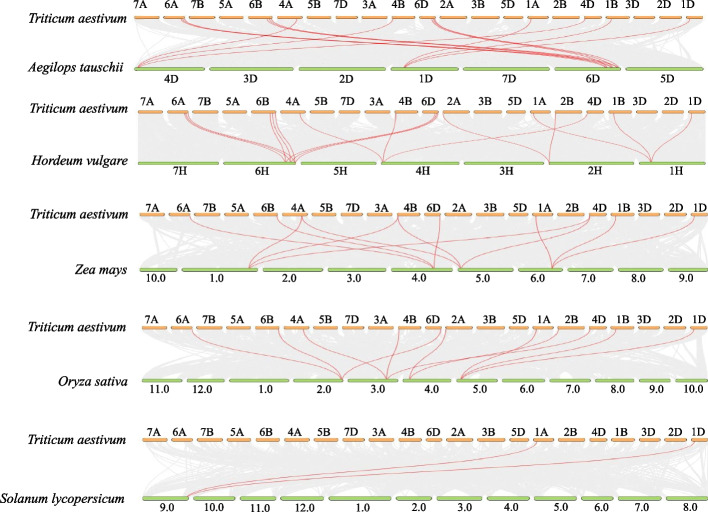
Synteny between *ETR* genes of wheat and five other representative plant species. Gray lines in the background indicate the collinear blocks between wheat and other plant genomes while the red lines indicate the syntenic *ETR* gene pairs

The 18 homoeologues of the six *TaETR* genes are found to be distributed unevenly over 4 chromosomes (chromosome 1, 2, 4 and 6) in each of the three subgenomes (A, B and D) of hexaploid wheat (Additional file 1: Fig. S1). Chromosomes 1, 2 and 4 of each subgenome contain one homoeologue while chromosome 6 of each subgenome consists of three homoeologues. To investigate the evolutionary relationships among the *TaETR*s, comparative syntenic maps of wheat and five representative plant species including one dicot (tomato) and four monocot (Tausch's goatgrass, barley, maize and rice) species were constructed (Fig. 1). A total of 17 *TaETR* genes showed syntenic relationship with those in barley while 15, 12 and 11 *TaETR* genes showed syntenic relationship with those in Tausch's goatgrass, maize and rice, respectively. In contrast, only two of the *TaETR* genes showed syntenic relationship with those in tomato. Duplication analysis of *TaETR*s revealed the presence of 18 pair of duplicated genes that are located on different chromosomes (Table S2). Furthermore, non-synonymous substitution rate (Ka), synonymous substitution rate (Ks) and Ka/Ks ratios for the 18 duplicated gene pairs were estimated to examine the selection pressure on *TaETR* genes during evolution (Table 2). The Ka/Ks ratios of all the duplicated *TaETR* gene pairs were < 1. Despite their high sequence conservation, the anticipated evolutionary time for *TaETR* duplication events ranges from 3.9 to 13.2 million years ago (Table 2).

**Table 2:** Ka/Ks ratios of duplicated *ETR* gene pairs of hexapolid wheat

Gene pairs	Ka^a^	Ks^b^	Ka/Ks	Mya^c^
*TaERS1A*/*TaERS1B*	0.003	0.128	0.022	9.832
*TaERS1A*/*TaERS1D*	0.002	0.123	0.017	9.461
*TaERS1B*/*TaERS1D*	0.005	0.103	0.048	7.891
*TaERS2A*/*TaERS2B*	0.011	0.083	0.133	6.362
*TaERS2A*/*TaERS2D*	0.008	0.076	0.108	5.809
*TaERS2B*/*TaERS2D*	0.005	0.093	0.056	7.171
*TaEIN4A*/*TaEIN4B*	0.006	0.172	0.037	13.232
*TaEIN4A*/*TaEIN4D*	0.013	0.143	0.088	10.997
*TaEIN4B*/*TaEIN4D*	0.009	0.125	0.073	9.598
*TaETR2A*/*TaETR2B*	0.008	0.085	0.097	6.535
*TaETR2A*/*TaETR2D*	0.003	0.051	0.067	3.908
*TaETR2B*/*TaETR2D*	0.008	0.064	0.128	4.958
*TaETR4A*/*TaETR4B*	0.018	0.077	0.228	5.955
*TaETR4A*/*TaETR4D*	0.014	0.053	0.259	4.044
*TaETR4B*/*TaETR4D*	0.021	0.052	0.401	3.986
*TaETR5A*/*TaETR5D*	0.020	0.081	0.247	6.268
*TaETR5A*/*TaETR5B*	0.035	0.094	0.373	7.240
*TaETR5D*/*TaETR5B*	0.036	0.082	0.436	6.328

^a^non-synonymous substitution rate

^b^synonymous substitution rate

^c^million years ago

### Structural organization of wheat ETR genes

**Fig. 2:**
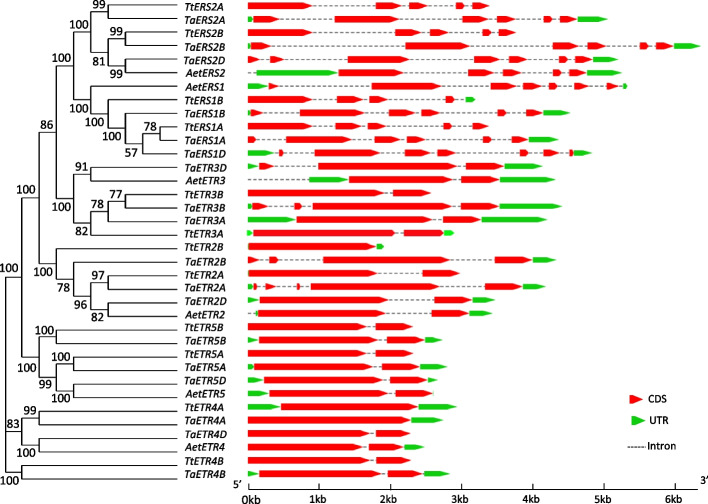
Exon/intron structural organization of *ETR* genes of hexaploid wheat and its progenitors *T. tugidum* and *Ae*. *tauschii*. The evolutionary relations among the *ETR* genes are represented by phylogenetic tree constructed with Maximum-likelihood method with 1000 bootstrap replicates using MEGA v10.1.8. Exon/intron structural organization were identified by aligning the genomic and coding sequences of each gene. The UTR regions are represented by the green bars, exons/coding sequence are shown by red bars and the connecting grey dashed lines represent the introns

We investigated the exon/intron structural organization of the *TaETR* genes by aligning their coding sequences against their corresponding genomic DNA sequences. The structural organization of the *TaETR* genes showed group specific exon/intron organizational pattern and they typically consist of one to seven exons and no intron to six introns (Fig. 2). The number of exons/introns varied in most of *TaETR* homoeologues derived from the three subgenomes. However, similar number of exon/introns were observed in *TaETR* genes derived from the D subgenome and those originated from one of the progenitors, *Ae*. *Tauschii*, except that the *AetERS2* and *AetETR3* genes exhibit two and one fewer exons, respectively. Whereas the *TaETR* genes derived from the A and B subgenomes have one or more exon/introns compared to those derived from *T*. *turgidum*. For example, six exons are present in the *TaERS1A* and *TaERS1B* homoeologues while five and four exons exist in the A and B genome copies of *ERS1* of *T. turgidum*, respectively (Fig. 2). Moreover, exon/intron structural analysis of the wheat, rice and Arabidopsis *ETR* genes revealed that exon/intron structural organization of the *TaETR*s is much closer to those of rice compared to the Arabidopsis *ETR* genes (Additional file 1: Fig. S2). Among the Arabidopsis five *ETR*s, only *ERS1* and *ETR1* showed exon/intron structural similarity with the wheat subfamily I *ETR* genes, *TaERS1* and *TaERS2*, respectively (Additional file 1: Fig. S2).

### Structural analysis of ethylene receptor proteins

**Fig. 3:**
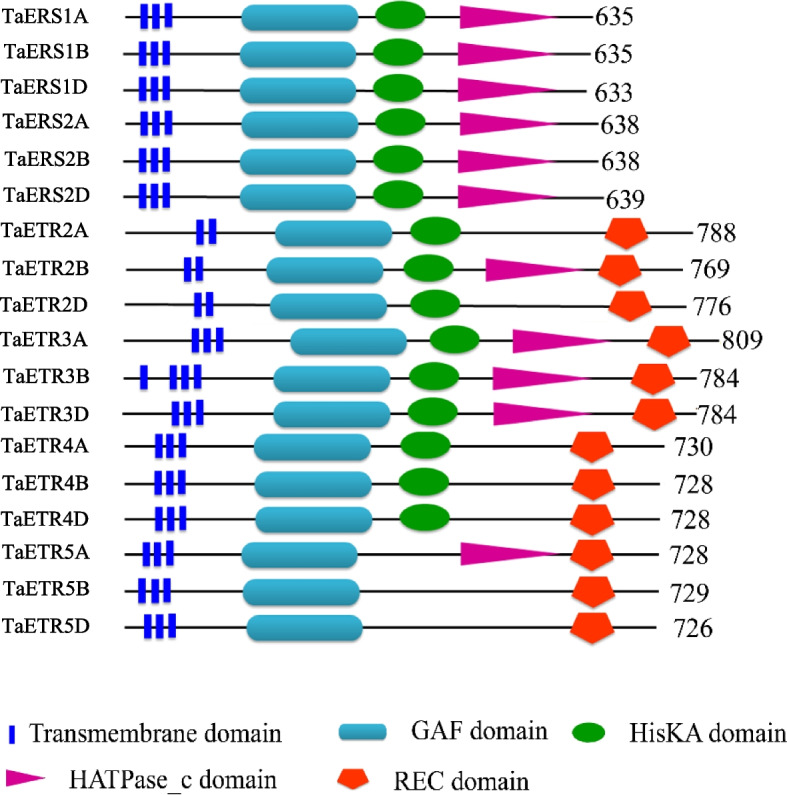
Schematic representation of the conserved domain organization of ETR proteins of hexaploid wheat. Conserved domains of the homologous proteins were analysed using SMART tool. GAF, cGMP phosphodiesterases/adenylyl cyclases/FhlA domain; HisKA, His Kinase A (phospho-acceptor) domain; HATPase_c, Histidine kinase-, DNA gyrase B-, and HSP90-like ATPase domain; REC, response regulator receiver domain

Structural analysis of the ETR proteins from hexaploid wheat and its progenitors revealed that they consist of a minimum of five and a maximum of seven domains (Fig. 3, Table S3). All the ETR proteins consist of GAF domain and a transmembrane region that contains two to three domains. Furthermore, transmembrane architecture analysis of the ETR proteins showed that they contain at least three conserved domains in the transmembrane region except for ETR2, which has only two domains at their N- terminal region. All the ETRs were then grouped into two subfamilies based on their conserved domain architecture; TaERS-type receptors were grouped in subfamily I while the TaETR-type receptors in subfamily II. The subfamily I receptors, which include TaERS1 and TaERS2, consist of HisKA domain followed by HATPase_c domain without a response regulator receiver (REC) domain. Whereas the subfamily II receptors, which include TaETR2, TaETR3, TaETR4 and TaETR5, consist of HisKA domain followed by either HATPase_c or REC domain except that TaETR2D, TaETR4, TaETR5B, TaETR5D, TtETR2, 4, TtETR5B and AetETR2, 3, 5 receptors lack the HATPase_c domain and the HisKA domain is absent in TaETR5A. Whereas the TaETR5B and TaETR5D receptors lack both the HATPase_c and HisKA domains. Moreover, members of the subfamily II receptors, ETR2 and ETR3, have additional putative signal peptide at their N- terminal region except for TtETR3A. Like the exon–intron structural organization, *ETR* gene family members with a relatively closer evolutionary relationship exhibit similarity in terms of their conserved domain organizations.

### Analysis of cis-regulatory elements in the promoters of TaETR genes

**Fig. 4:**
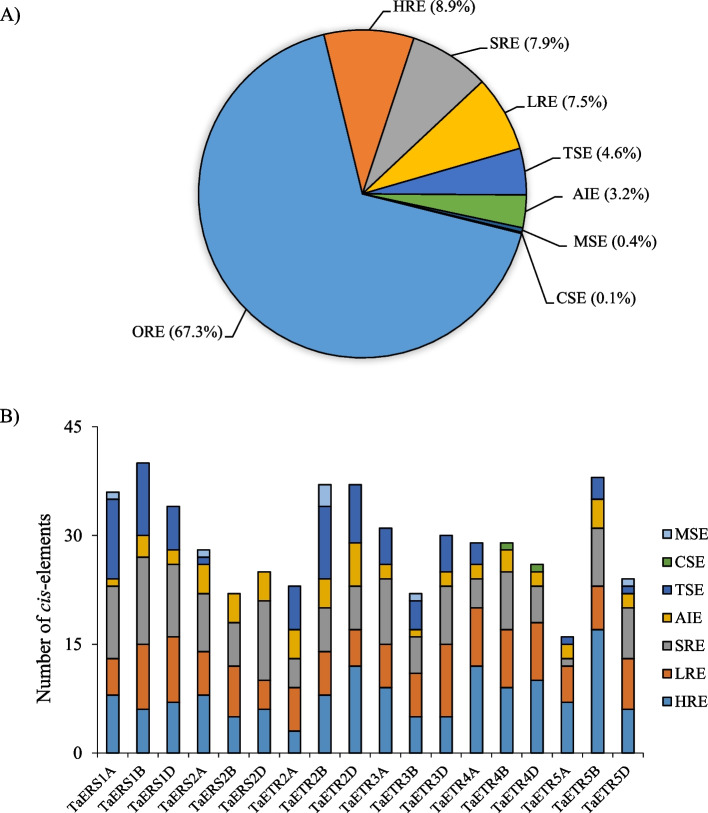
Cis-regulatory elements in the promoters of *ETR* genes of hexaploid wheat. Percentage of cis-elements that belong to the eight functional groups (**A**), and number of cis-elements in the promoter of each *ETR* gene per each functional group (**B**). ORE, other responsive elements; HRE, hormone responsive elements; SRE, stress responsive elements; LRE, light responsive elements; TSE, tissue specific elements; AIE, anaerobic induction elements; MSE, metabolism specific elements; CSE, cell cycle specific elements

To identify the cis-regulatory elements, a 1.5 kb sequence upstream of the start codon of each *TaETR* gene was analyzed using PlantCARE database [59]. Our analysis revealed a total of 67 cis-regulatory elements with more than one occurrence in most of *TaETR* promoters (Table S4). Excluding the core promoter elements such as TATA and CAAT box, the remaining cis-regulatory elements are categorized into eight groups based on their functional association (Fig. 4, Table S4). These groups are hormone responsive elements (HRE), stress responsive elements (SRE), light responsive elements (LRE), tissue specific elements (TSE), anaerobic induction elements (AIE), metabolism specific elements (MSE), cell cycle specific elements (CSE) and other responsive elements (ORE). The ORE group consists of the highest number (67.3%) of cis-regulatory elements mainly with unknown function followed by HRE (8.9%), SRE (7.9%), LRE (7.5%), TSE (4.6%), AIE (3.2%), MSE (0.4%) and CSE (0.1%) (Fig. 4A). Of the 18 *TaETR* homoeologues, the promoter of *TaETR5A* consists of the least number of cis-regulatory elements (16) while the promoter of *TaERS1B* consists of the highest number of cis-regulatory elements (40) (Fig. 4B).

Promoter of each *TaETR* homoeologue has one or more cis-regulatory elements from each group except the CSE and MSE groups (Table S4). The CSE cis-regulatory elements are present only in the promoters of *TaETR4B* and *TaETR4D*. The MSE cis-regulatory elements that are involved in the regulation of zein metabolism are present in the promoters of *TaERS1A*, *TaERS2A*, *TaETR3B* and *TaETR2B* while those involved in the regulation of flavonoid biosynthesis are found only in the promoter of *TaETR5D* (Table S4). Among the six types of hormone responsive cis-regulatory elements identified in the *TaETR* gene promoters, the abscisic acid-responsive (ABRE) and methyl jasmonate-responsive (CGTCA, TGACG and JERE) elements are highly overrepresented followed by gibberellin responsive (P-box and GARE), ethylene-responsive (ERE), auxin-responsive (TGA-element) and salicylic acid responsive (TCA-element) elements (Table S4). Promoters of all *TaETR* genes consist of a cis-regulatory element that is responsible for anaerobic (ARE) or anoxic (GC-motif) induction. Among the stress responsive cis-regulatory elements, promoters of the *TaETR* genes are highly overrepresented with wound responsive elements (W box, WRE3 and WUN motifs) followed by drought (MYB binding sites), low temperature (LTR), dehydration (DRE1 and DRE core), oxidative (as-1) and defense (TC-rich repeats) responsive elements (Table S4).

### Expression patterns of TaETR genes in different tissues during development

**Fig. 5:**
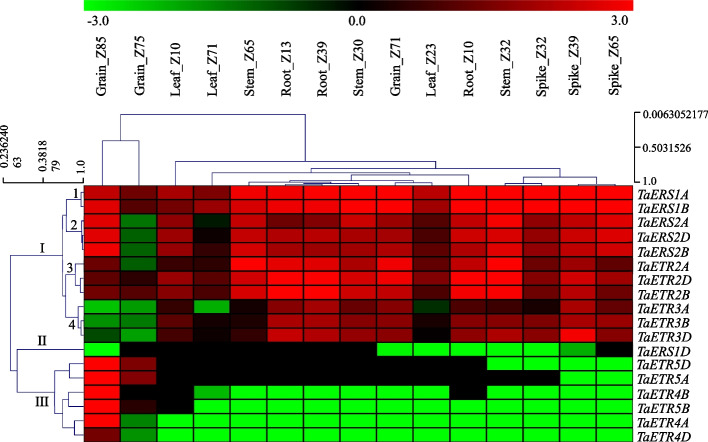
Expression patterns of *TaETR* gene family members in different tissues at different developmental stages. The transcript per million (TPM) values extracted from publicly available RNA-seq data were log_2_ transformed and converted to expression values in log_2_ fold changes shown by the negative and positive numbers and the color scale bar at the top of heat map; higher and lower expression levels of the respective genes are represented by red and green colors, respectively

To examine the expression patterns of the *TaETR* genes in different tissues and developmental stages, transcriptome data of root, leaf, stem, spike and grain tissues at different developmental stages were retrieved from expVIP [64]. Expression data of the 18 *TaETR* homoeologues showed their distinct tissue and stage specific expression patterns except *TaERS1A* and *TaERS1B*, which exhibited an increase in their expression level with development irrespective of tissue type (Fig. 5). Based on their expression patterns, the *TaETR* homoeologues are grouped mainly into three groups. The homoeologues in group I are further categorized into four subgroups. Homoeologues in subgroup 1 (*TaERS1A* and *TaERS1B*) are highly expressed in all tissues at all developmental stages while those in subgroup 2 (*TaERS2A*, *TaERS2B* and *TaERS2D*) and 3 (*TaETR2A, TaETR2B* and *TaETR2D*) showed enhanced expression in all tissues at all developmental stages considered except those in subgroup 2 are repressed in grains at Z75 and homoeologues in subgroup 3 showed lower levels of expression in Z85 grains than those in subgroup 2. The homoeologues in subgroup 4 (*TaETR3A, TaETR3B* and *TaETR3D*) exhibited high levels of expression in the root and spike tissues with minimal expression detected in all other tissues. Group II consists of only one gene, namely *TaERS1D*, which is either minimally expressed or not expressed in all tissues irrespective of developmental stages. Group III consists of the homoeologues of *TaETR4* and *TaETR5*, whose expression is either minimal or not detected in all tissues irrespective of developmental stages except that these genes, specifically the *TaETR4A* and *TaETR4B* homoeologues, exhibited high level of expression in grains at Z85 stage.

All the *TaETR* genes exhibited high expression level in the grain at least in one of its developmental stages except that they exhibit either minimal or no expression at Z75 stage. They are also upregulated in the root, stem and spike tissues at different stages of development except that *TaETR4* and *TaETR5* homoeologues and one of the homoeologues of *TaERS1* (*TaERS1D*) exhibit either minimal or no expression in these tissues (Fig. 5). The expression levels of these genes were also either repressed or not detected in the leaf tissues at all developmental stages. The other *TaETR*s showed enhanced expression levels in leaf tissues at all developmental stages except the expressions of *TaERS2* and *TaETR3* are supressed at Z71 and Z23 stages (Fig. 5).

### Changes in morphological traits under abiotic stress conditions and in response to ethylene

#### Morphological traits under drought and salinity stress and in response to ethylene

**Fig. 6:**
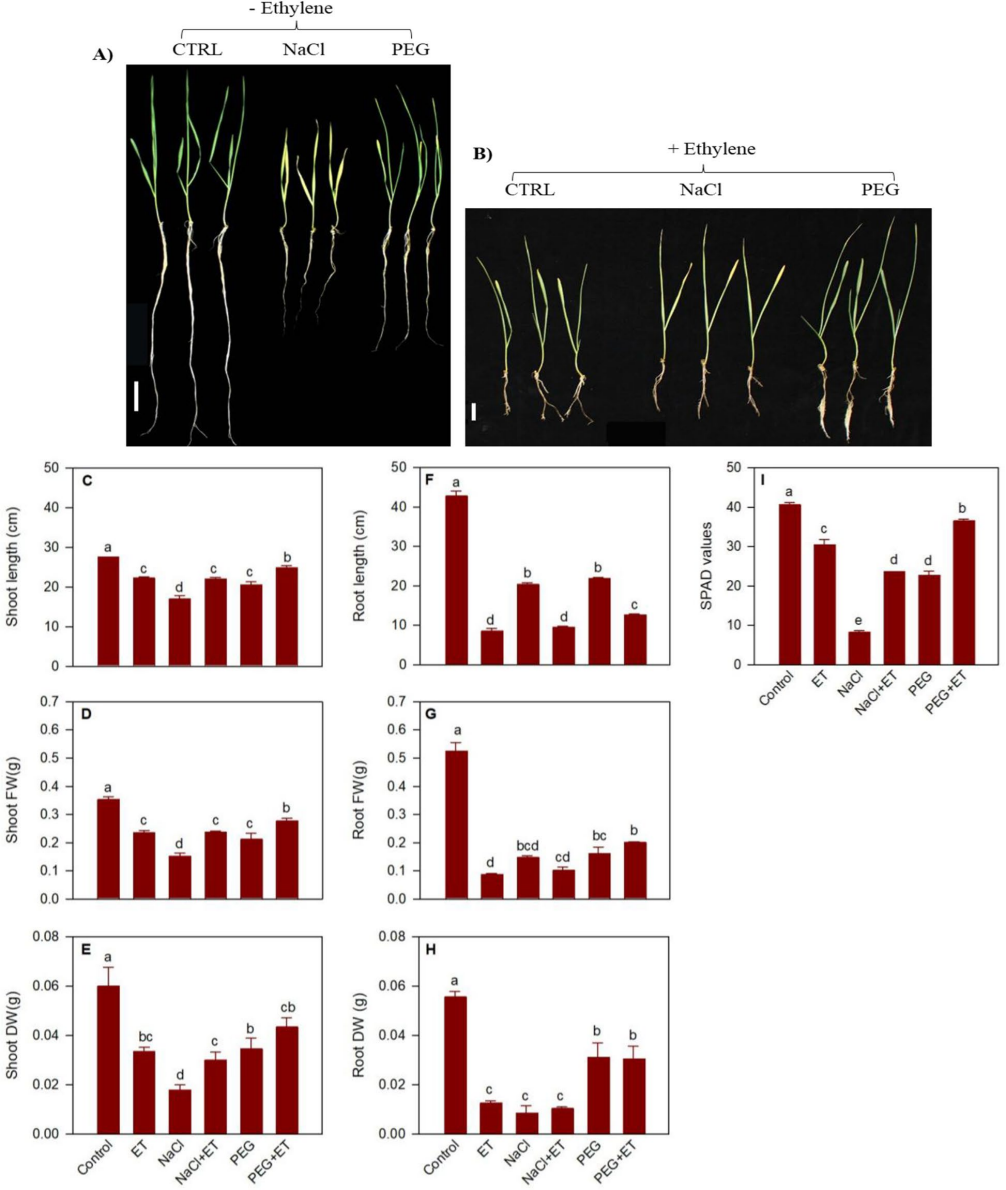
Morphological traits and chlorophyll content. Wheat seedlings exposed to sanity and drought stress with or with out ethylene treatment (**A**, **B**). Changes in shoot and root length (**C**, **F**), and fresh weight (FW; **D**, **G**) and dry weight (DW; **E**, **H**), and leaf chlorophyl content (I) of seedlings in response to treatment with ethylene, and salinity and drought stress with or without prior ethylene treatment. Data are means ± SE, *n* = 3, where n represents a group of nine seedlings. Different letters indicate statistically significant differences among samples at *P* < 0.05 (LSD test). Scale bars are 1 cm each

Treatment with exogenous ethylene led to reduction in shoot length (19%), fresh weight (33%) and dry weight (44%) compared to the control untreated seedlings (Fig. 6A-E). Salinity stress for 3 days caused more reduction in shoot length (38%), fresh weight (57%) and dry weight (70%) (Fig. 6A-E). However, treatment of the seedlings with ethylene prior to applying the stress reversed the effect of salinity stress on shoot length, fresh weight and dry weight of seedlings to the level observed in control seedlings treated with ethylene alone. Relative to that observed in control seedlings, exogenous ethylene and salinity stress caused a 25% and 80% decrease in leaf chlorophyll content, respectively (Fig. 6I). However, ethylene treatment of seedlings before subjecting them to salinity stress led to a 65% increase in chlorophyll content compared to salinity stressed seedlings with no ethylene treatment (Fig. 6I). With respect to root tissue, exogenous ethylene inhibited root length (80%), fresh weight (82%) and dry weight (77%) compared to the control untreated seedlings (Fig. 6A, B, F–H). Salinity stress also inhibited root growth; it caused reductions in root length (53%), fresh weight (72%) and dry weight (85%) (Fig. 6 A, B, F–H). No significant difference in root growth was evident between control seedlings treated with ethylene and salinity stressed seedlings with or without prior treatment with ethylene except that root length was less affected by salinity stress involving no exogenous ethylene (Fig. 6G-H).

Drought stress of seedlings for 3 days inhibited shoot growth in terms of shoot length (25%), fresh weight (40%) and dry weight (43%) compared to the control counterparts (Fig. 6A-E). The magnitude of its effect on shoot growth was similar to that caused by exogenous ethylene. Treatment of seedlings with ethylene prior to subjecting them to drought stress, however, promoted shoot growth compared to that observed in drought stressed seedlings with no prior ethylene treatment; it caused increases in shoot length (17%), fresh weight (24%) and dry weight (21%). Drought stress also reduced chlorophyll content (44%) compared to control seedlings. Interestingly, treatment of seedlings with ethylene prior to drought stress increased chlorophyll content by 38% and 17% compared to that observed in drought stressed seedlings with no prior ethylene treatment and control seedlings treated with ethylene, respectively (Fig. 6I).

Drought stress also inhibited root growth; it reduced root length (49%), fresh weight (70%) and dry weight (44%) compared to that observed in control seedlings (Fig. 6A, F–H). Treatment of seedlings with ethylene prior to applying drought stress led to further decrease in root length (70%), but the roots of these seedlings were 32% longer than that observed in control seedlings treated with ethylene alone (Fig. 6B, F). No significant differences in root fresh weight and dry weight were observed between drought stressed seedlings treated with or without prior treatment with exogenous ethylene. However, drought stressed seedlings with prior ethylene treatment exhibited higher root fresh weight (57%) and dry weight (60%) than the control seedlings treated with ethylene (Fig. 6G-H).

#### Morphological traits under heat and cold acclimation and in response to ethylene

**Fig. 7:**
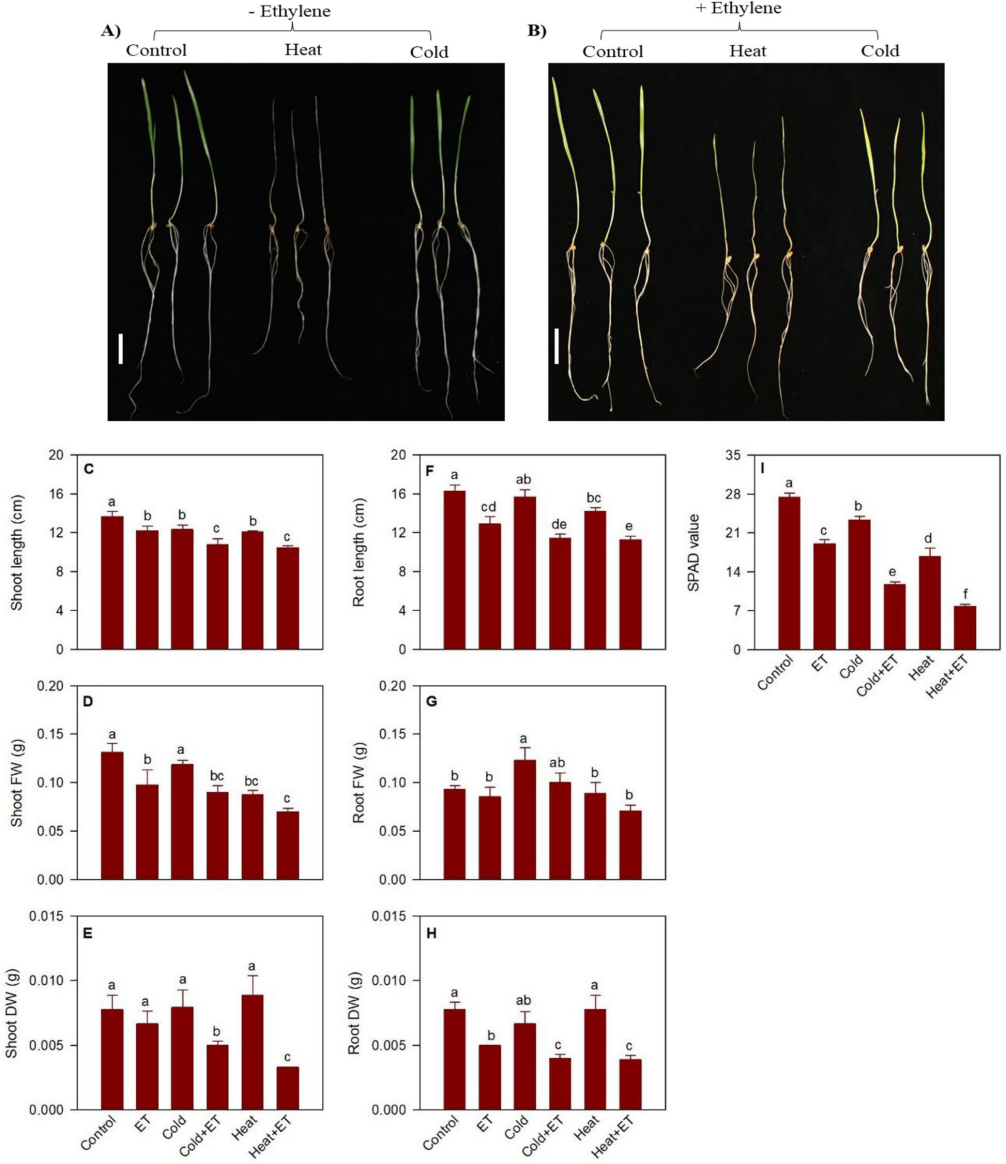
Morphological traits and chlorophyll content. Wheat seedlings exposed to cold and heat stress with or with out ethylene treatment (**A**, **B**). Changes in shoot and root length (**C**, **F**), and fresh weight (FW; **D**, **G**) and dry weight (DW; **E**, **H**)), and leaf chlorophyl content (**I**) of seedlings in response to treatment with ethylene, and cold and heat stress with or without prior ethylene treatment. Data are means ± SE, *n* = 3, where n represents a group of nine seedlings. Different letters indicate statistically significant differences among samples at *P* < 0.05 (LSD test). Scale bars are 3 cm each

Heat stress inhibited shoot growth, and this effect was exacerbated by exogenous ethylene. Heat stress reduced shoot length (12%) and fresh weight (33%) with no apparent effect on its dry weight compared to that observed in control seedlings (Fig. 7A-E). Treatment of seedlings with ethylene prior to heat stress led to further reduction of shoot length (24%), fresh weight (47%) and dry weight (57%) compared to that exhibited by the control seedlings. Leaf chlorophyll content in heat stressed seedlings was reduced by 39% compared to that detected in the corresponding control seedlings, and ethylene treatment prior to applying the heat stress reduced chlorophyll content further to 47% of that observed in heat stressed seedlings with no prior ethylene treatment (Fig. 7I). Heat stress also inhibited root elongation (12.6%) with no significant effect on root fresh and dry weight (Fig. 7A, B, F–H). Prior treatment with ethylene of heat stressed seedlings led to further reduction in root length (40%) and dry weight (50%) with no significant effect on its fresh weight compared to that observed in heat stressed seedlings that received no prior treatment with ethylene.

No significant effect of cold acclimation was apparent on shoot fresh and dry weight as well as root length and dry weight of seedlings compared to their control counterparts (Fig. 7A-E, F–H). However, shoot length was reduced by 10% while root fresh weight was increased by 24% (Fig. 7C, G). Prior treatment with ethylene before cold acclimation further reduced the shoot length (21%), fresh weight (31%) and dry weight (36%) as well as root length (30%) and dry weight (48%) compared to that observed in the control seedlings (Fig. 7C-E, F, G). However, no significant difference in root fresh weight was observed between cold acclimated seedlings with and without prior to ethylene treatment (Fig. 7F). Leaf chlorophyll content in cold acclimated seedlings was reduced by 15% compared to that observed in the control seedling (Fig. 7I), and ethylene treatment further decreased leaf chlorophyll (57%) in cold acclimated seedlings compared to the control counterparts.

## Expression analysis of *TaETR* genes under abiotic stress conditions

### Expression of TaETR genes in response to salinity and drought stress

**Fig. 8:**
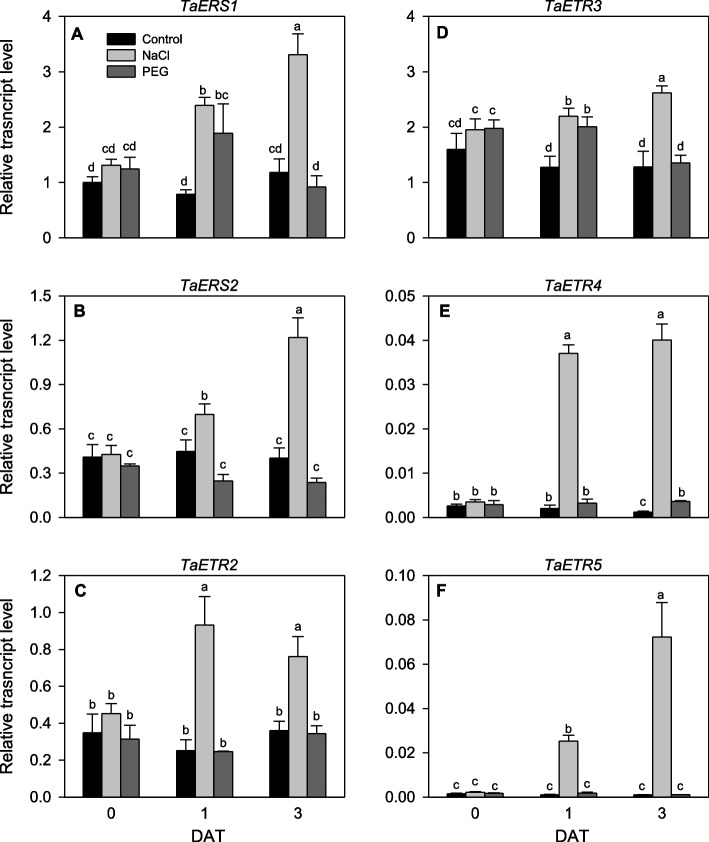
Expression patterns of *TaETR* genes in response to salinity or drought stress. Relative transcript levels of the *TaETR* genes (**A-F**) in seedlings at 0, 1 and 3 days after treatment with salinity or drought stress (DAT). Transcript levels of the *TaETR*s were determined using *Ta18S rRNA* as the reference gene and expressed relative to the transcript levels of *TaERS1* in the control samples at 0 DAT, which were arbitrarily set to a value of 1. Data represents means of three independent biological replicates ± SE. Different letters indicate statistically significant differences among samples at *P *< 0.05 (LSD test)

Overall, the *TaERS1* and *TaETR3* genes exhibited the highest levels of expression in the control unstressed seedlings while *TaETR4* and *TaETR5* showed low expression levels (Fig. 8). Salinity stress for 1 day upregulated (1.6- to 26.3-fold) all the six *TaETR* genes. Likewise, extending the duration of salinity stress to 3 days led to upregulation of all the *TaETR* genes (2.0- to 73.4-fold). The expression levels of *TaERS1* (2.4-fold) and *TaETR3* (1.6-fold) were induced at 1 day after drought stress and that of *TaETR4* (3.0-fold) at 3 days after drought stress compared to the respective controls (Fig. 8). The expression levels of these three genes at the other time points of drought stress and that of *TaERS2*, *TaETR2* and *TaETR5* at all time points were similar to that observed in the corresponding control seedlings not exposed to drought stress.

### Expression of TaETR genes in response to cold and heat stress

**Fig. 9:**
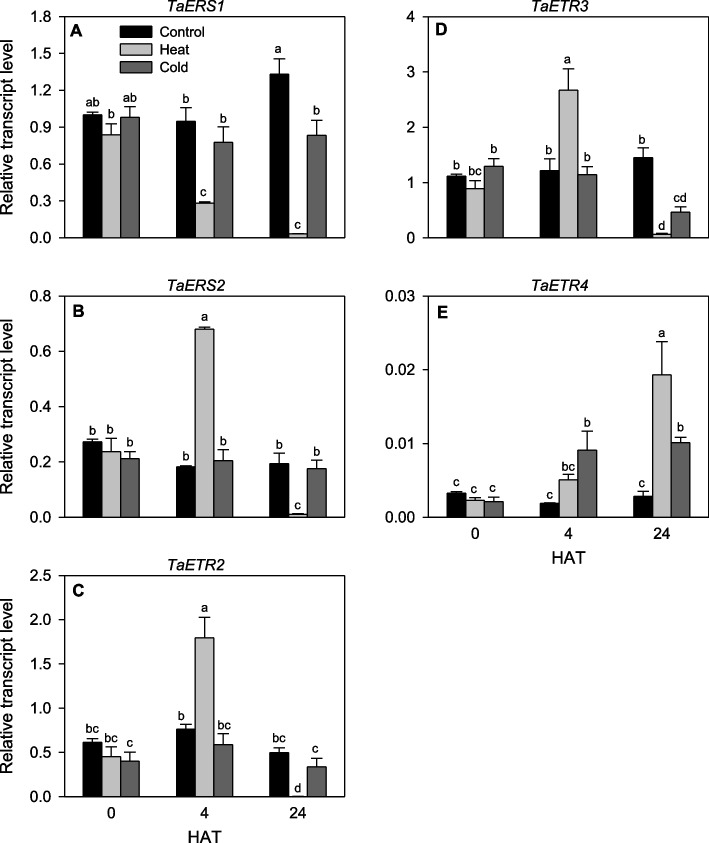
Expression patterns of *TaETR* genes in response to heat and cold acclimation. Relative transcript levels of the *TaETR* genes (**A-E**) in seedlings at 0, 4 and 24 h after treatment with heat or cold (HAT). Transcript levels of the *TaETR*s were determined using *Ta18S rRNA* as the reference gene and expressed relative to the transcript levels of *TaERS1* in the control samples at 0 DAS, which were arbitrarily set to a value of 1. Data represents means of three independent biological replicates ± SE. Different letters indicate statistically significant differences among samples at *P* < 0.05 (LSD test)

Heat stress for 4 h induced the expression levels of three *TaETR* genes, namely *TaERS2*, *TaETR2* and *TaETR3* (over twofold) compared to that observed in the unstressed control seedlings. By 24 h after heat stress, the expression of these genes was reduced to very low level. The expression level of *TaETR4* was induced by heat stress for 4 h (2.7-fold) or 24 h (6.8-fold) (Fig. 9E). In contrast, the expression of *TaERS1* was repressed in response to heat stress for 4 h (3.4-fold), and extending the duration of heat stress to 24 h led to further repression of its expression (42-fold) compared to that observed in the unstressed control seedlings. Expression levels of all the *TaETR* genes remained unaffected in response to cold acclimation for 4 and 24 h except that the expressions of *TaERS1* (1.6-fold) and *TaETR3* (3.1-fold) were repressed at 24 h after cold acclimation while that of *TaETR4* was upregulated by over 3.6-fold at 4 and 24 h after cold acclimation, respectively, compared to that observed in the respective control seedlings (Fig. 9). No expression of *TaETR5* was detected in the seedlings irrespective of the type or duration of treatment.

### Expression of TaETR genes in response to waterlogging

**Fig. 10:**
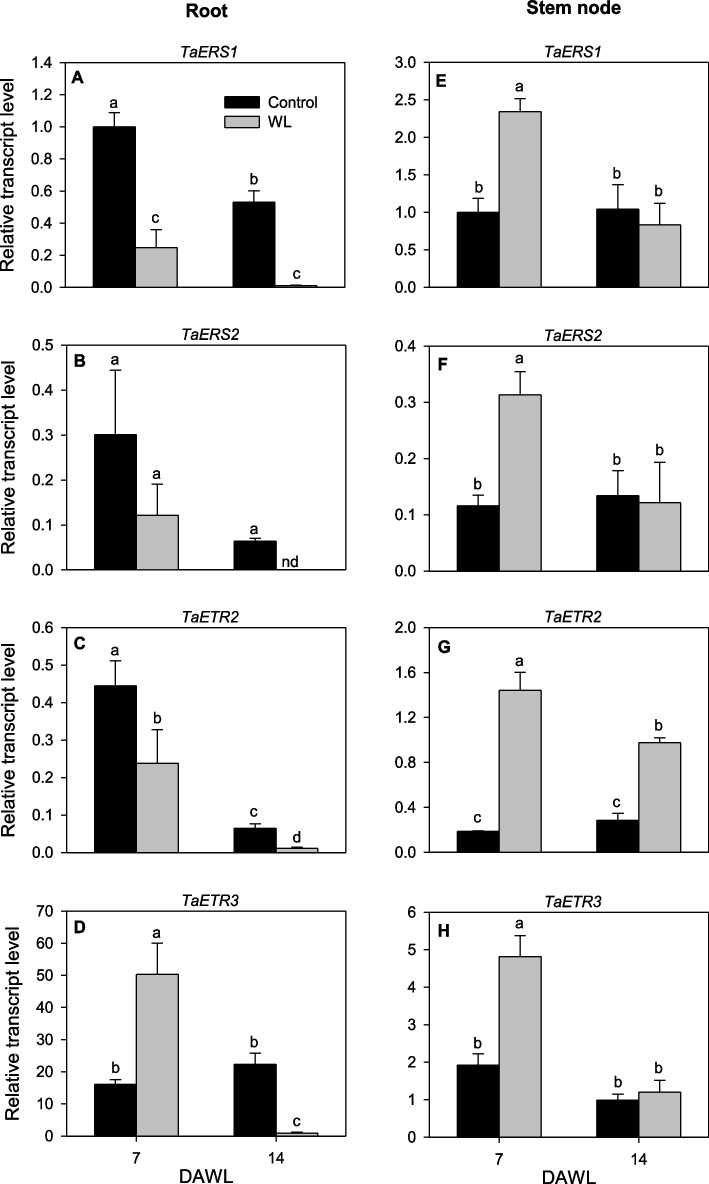
Expression patterns of *TaETR *genes in response to waterlogging. Relative transcript levels of the *TaETR* genes in root (**A-D**) and stem node (**E-H**) tissues at 7 and 14 days after waterlogging (DAWL). Transcript levels of the *TaETR*s in each tissue were determined using *Ta18S rRNA* as the reference gene and expressed relative to the transcript levels of *TaERS1* in the respective control samples at 7 DAWL, which were arbitrarily set to a value of 1. Data represents means of three independent biological replicates ± SE. Different letters indicate statistically significant differences among samples at *P *< 0.05 (LSD test). Changes in root and shoot morphological traits in response to waterlogging are as reported previously [94]

Given that ethylene plays significant role in enhancing plant adaptive responses to waterlogging stress, we examined the expression patterns of *TaETR* genes in wheat root and stem node tissues of wheat plants waterlogged for 7 and 14 days. Four *TaETR* genes, namely *TaERS1*, *TaERS2*, *TaETR2* and *TaETR3*, showed differential expression in root and stem node tissues in response to the water waterlogging (Fig. 10) while no expression of *TaETR4* and *TaETR5* was detected irrespective of tissue and treatment. The expression levels of *TaERS1*, *TaERS2* and *TaETR2* in the root tissue were repressed by waterlogging for 7 and/or 14 days (Fig. 10A, C, and E). In contrast, the expression level of *TaETR3* was induced (3.1-fold) by waterlogging for 7 days although it was repressed (25-fold) in response to waterlogging for 14 days (Fig. 10D). All the *TaETR* genes in the stem node tissue showed upregulation (2.3- to 7.7-fold) in response to waterlogging for 7 days (Fig. 10E-H). However, their expression levels were not affected by 14 days of waterlogging except that *TaETR2* exhibited upregulation (3.4-fold).

## Discussion

Ethylene regulates a wide range of plant growth and developmental process and mediates plant responses to biotic and abiotic stresses [68, 70]. These roles of ethylene involve ETRs, proteins that perceives it and serve as the first entry point of its signal transduction. To regulate ethylene signaling in tissue/stage specific manners and in response to different environmental cues, plants have evolved to have multiple ETRs encoded by gene family members with distinct transcriptional regulatory mechanisms [41, 43]. Recent advances in whole-genome sequencing technologies have provided opportunities for genome-wide identifications and characterizations of genes controlling important crop traits such as the *ETR*s. Previous studies showed that diploid species such as Arabidopsis, tomato and rice each has five *ETR*s [39, 41, 42]. The identification of six *ETR* gene family members in hexaploid wheat (18 homoeologues) as well as in tetraploid *T*. *turgidum* (12 homoeologues) and diploid *Ae*. *tauschii* genomes suggest that the number of *ETR* gene family members and their homoeologues varies with plant species and genome duplication events. Consistently, the allotetraploid soybean genome has been shown to have a total of 11 ETRs; four *EIN4*, two *ETR1*, *ERS1* and *ETR2*, and one *ERS2* gene copies resulted from duplication events [43, 71]. The prevalence of varied number of *ETR*s in different plant species may represent the hierarchy in functional complexity of ethylene perception and cellular responses to developmental events and external stimuli.

Structural organization of gene family members provides insights into the evolutionary mechanisms underlying their genesis [72]. Exon/intron structural divergence of genes involves three mechanisms including exon/intron gain or loss, insertion or deletion, and exonization or pseudoexonization [73]. Since the large number of introns observed at the early stages of eukaryotic development tended to decrease over time, introns are believed to play key roles in plant evolution [74]. However, an increase in the number of introns has also been reported with its significance of inducing evolution mediated new gene functions [75–77]. Consistently, gain of introns was observed in A and B genome copies of *TaETR*s compared to the corresponding genes in *T*. *turgidum*. In addition, the D genome copies of *TaERS2* and *TaETR3* exhibit extra introns compared to those in *Ae*. *Tauschii*. These results may support the hypothesis that homoeologous recombination events might have occurred among *ETR* genes of the progenitors during evolution of hexaploid wheat.

Despite the architectural divergence between *ETR*s of hexaploid wheat and its progenitors, their corresponding proteins exhibited highly conserved functional domains as observed for members of the subfamily I ETRs, namely ERS1 and ERS2, implying absence of significant functional divergence among the ETRs of wheat and its progenitors. In support of this, similar structural domains exist in ERS1 of soybean and Arabidopsis [43]. Subfamily II ETRs (ETR 2, 3, 4 and 5) of hexaploid wheat and its progenitors are also highly conserved. Notably, TaETR2B, TaETR3 and TaETR5A possess HATpase_c domain, which represents histidine kinase activity of ETR-1 receptors [78], highlighting the role of this domain in ethylene perception and downstream signaling. Despite these reports, specific roles of the loss or gain of functional domains of ETRs in regulating the perception of ethylene and its downstream signal transduction are unknown, emphasizing the need of further studies in this regard.

Cis-regulatory elements in promoter sequences play crucial roles in regulating gene expression [79]. Therefore, the observation of distinct distribution of cis-regulatory elements in the promoters of *TaETR*s may explain their roles in regulating the unique spatio-temporal expression patterns of the *ETR* genes during wheat plant development and in response to environmental cues. The presence of hypoxia specific anaerobic induced elements (ARE and GC-motif) and their frequency in promoter sequence of a target gene are indicators of its role in mediating adaptive response of plants to waterlogging/flooding stress [80]. Our analysis revealed that promoter sequences of *TaERS1*, *TaERS2*, *TaETR2*, and *TaETR3* consist of ARE and/or GC-motif in abundance, thus, upregulation of these *TaETR* genes in the stem nodes of waterlogged plants may indicate importance of the frequency/distribution of the two motifs in transcriptional activation of the specific *TaETR*s in response to waterlogging. In contrast, promoter sequences of *TaETR4* and *TaETR5* genes consist of only fewer ARE motifs and lack the GC-Motif (Table S4), and this may explain the absence of any detectable transcripts of the two genes in the stem node tissues of waterlogged plants. Consistently, *alcohol dehydrogenase* gene family members of wheat that possess ARE cis-regulatory elements in their promoters showed enhanced expression under waterlogging conditions [81]. Furthermore, *ETR* genes of rice have been shown to exhibit differential expression in response to submergence [37, 82].

Our study showed that salinity stress results in reduction in shoot and root growth and leaf chlorophyll content as observed previously [20, 83]. However, treatment of seedlings with ethylene prior to their exposure to salinity stress reversed adverse effects of the stress on shoot but not on root growth, reflecting the role of ethylene in conferring salinity stress tolerance in a tissue specific manner. Consistently, inhibition of ethylene biosynthesis and silencing of *ETR* gene led to reduced tolerance to salt stress in *Solanum chilense* and alfalfa [20, 83]. Therefore, upregulation of all six *TaETR*s in response to salinity stress reflects their participation in ethylene mediated modulation of wheat tolerance to salt stress. Previous studies demonstrated that ABRE, G-box and TGA *cis-regulatory*-elements play important role in transcriptional regulation of salt responsive genes in plants [84, 85], and these motifs are present in the promoter sequences of *TaETR*s. For example, the promoter sequence of *TaETR5* exhibited the highest number of ABRE and G-box elements while that of *TaETR3* consists fewest number of these motifs. Consequently, *TaETR5* exhibited strong transcriptional induction in response to salt stress whereas *TaETR3* showed relatively weak induction. Our data therefore implicate the importance of distribution/frequency of ABRE, G-box and TGA motifs in the promoter sequences of *TaETR*s to control their expression under salinity stress. It has been reported previously that ethylene mediates salinity stress tolerance via increasing leaf chlorophyll content, reducing ROS generation, and enhancing antioxidant enzyme activity and proline accumulation [20]. However, the mechanisms underlying the role of ethylene in regulating these physiological events remain to be elucidated.

Drought stress also inhibited seedling growth and caused reduction of chlorophyll content. However, ethylene treatment prior to drought stress significantly improved shoot but not root growth of seedlings as observed in salinity stressed seedlings. Similarly, exogenous ethylene has been shown to improve wheat survival rate under drought condition through enhancing shoot biomass [19]. A previous study in tomato also demonstrated that exogenous ethylene induces drought tolerance via reducing transpiration rate/water loss [86]. Such an effect along with the observed increase in chlorophyll content may imply the role of ethylene in enhancing photosynthetic capacity under drought conditions. Thus, upregulation of *TaERS1*, *TaETR3* and *TaETR4* in response to drought stress implicate the role of specific *TaETR*s in regulating ethylene signaling and thereby drought tolerance in wheat. Induction in the expression of these *TaETR* genes under drought stress is closely associated with presence and distribution of drought responsive *cis-regulatory*-elements in their promoter sequences. For example, the promoter region of *TaERS1*, which exhibits strong induction in its expression under drought stress, consists of the highest number of MYB recognition and drought responsive (DRE1) core motifs while absence of any detectable expression of *TaETR5* in response to drought can be attributed to the very few DRE1 cis-regulatory elements present in its promoter sequence.

Our study showed that heat stress adversely affects shoot length and fresh weight, leaf chlorophyl content, and root length but not shoot dry weight and root fresh and dry weights. Prior treatment with ethylene of heat stressed seedlings, however, led to further suppression of all the traits studied. These effects of ethylene contrasts with that observed under salinity or drought stress, suggesting ethylene’s role as a negative regulator of heat tolerance of wheat seedlings. Given that heat stress inhibits photosynthesis [10], further reduction in leaf chlorophyll content due to prior treatment of heat stressed seedlings with ethylene may represent the synergistic roles of ethylene and heat stress in limiting photosynthesis and therefore seedling growth. In agreement with these results, exogenous ethylene further lowers the level of antioxidants such as ascorbate under heat stress conditions [87]. Thus, upregulation of four *TaETR* genes within 4 h of heat stress might indicate their role in inducing ethylene signaling and thereby limiting wheat growth under elevated temperature. Despite this observation, no cis-regulatory-element directly involved in heat response such as heat shock elements was detected in the promoter sequences of *TaETR* genes. However, our analysis showed that the *TaETR* promoters harbour other cis-regulatory elements such as G-box, ABRE and W-box, as well as MYB recognition sites that are implicated in the regulation of heat stress tolerance through cooperating with heat shock elements [88–90]. Consistently, *TaERS1*, *TaETR5* and *TaETR3*, which consist of two or more of the four motifs, showed enhanced expression under heat stress.

Wheat seedlings exhibited minimal changes in growth and leaf chlorophyl content under low temperature conditions. Thus, inhibition of seedling growth and reduction of chlorophyl content when cold acclimation is combined with ethylene suggest that ethylene negatively influence cold tolerance in wheat. Similarly, exogenous ACC, a precursor of ethylene, or ethephon has been shown to decrease tolerance to cold stress in *Hevea brasilien*sis and soybean seedlings [91, 92]. Therefore, upregulation of *TaETR4* in response to cold acclimation suggests its role in mediating ethylene perception and inducing downstream signalling and cold acclimation-mediated repression of seedling growth. Although the promoters of *TaERS1*, *TaERS2*, *TaETR3*, and *TaETR5* genes consist of a high number of core cold-responsive motifs (LTR and DRE core), no induction in their expression level was observed under cold acclimation. However, *TaETR4*, which consists of high number of secondary cis-regulatory elements such as MYB binding site and ABRE along with the core cold-responsive motifs in its promoter, exhibited strong induction in its expression in response to cold treatment. These observations suggest the requirement of not only cold responsive cis-elements but also supportive secondary cis-regulatory elements in regulating the expression of *TaETR*s under cold acclimation. The growth inhibitory effect of ethylene under cold stress may also be linked to repression of C-repeat Binding Factor/DRE Binding Factor (CBF/DREB) transcriptional regulatory cascade as previously reported [72, 93]. It has been shown previously that *ETR*s of rice are upregulated under submergence condition [37, 94]. Likewise, we observed upregulation of *TaETR3* in the root and four *TaETR*s (*TaERS1*, *TaERS2*, *TaETR2* and *TaETR3*) in the stem node tissues of waterlogged plants. These results suggest distinct and overlapping tissue specificity of *TaETR*s in regulating ethylene perception and signalling to induce adaptive morphological and anatomical responses and thereby confer tolerance against waterlogging stress [71].

Despite the observation of close associations between distribution of cis-regulatory elements and expression patterns of *TaETR* genes in response to the abiotic stress factors, we were unable to establish a clear connection between tissue specificity of the expression of *TaETR* genes during wheat development and distribution of cis-regulatory elements in their respective promoter sequences. This might be due to availability of limited number of well-characterized tissue specific cis-regulatory elements in plants.

## Conclusions

The present study identified and characterized the genomic, structural, evolutionary and molecular characteristic features of six *ETR* genes and the respective 18 homoeologues of hexaploid wheat. The structures of these genes and functional domains of their corresponding proteins appeared to be conserved among themselves and with their orthologs in the tetraploid and diploid progenitors of hexaploid wheat. However, the distinct distribution pattern of cis-regulatory elements in the promoter sequences of *TaETR* genes may indicate their differential molecular functions in regulating the expression of *TaETR* genes and thereby influence plant developmental processes and its response to diverse environmental stresses. Since this study was limited to in silico analyses of the candidate genes and investigating their expression patterns across developmental stages and in response to multiple abiotic stresses, it provided preliminary insights into the potential function of wheat *ETR* genes. Therefore, further validation of these findings through molecular and genetic studies is crucial to elucidate their physiological function for enhancing our understanding of ethylene signaling in wheat and there by applying these genes in stress adaptive breeding programs.

## Supplementary Information

Additional file 1: Fig. S1. Chromosomal location of *ETR* gene family members of hexaploid wheat. Fig. S2. Evolutionary relationship and exon/intron structural organization of the *ETR* genes in hexaploid wheat, Arabidopsis and rice.

Additional file 2: Table S1. Primers sequences used for gene expression analysis

Additional file 3: Table S2. Duplication events of *ETR* gene family members in hexaploid wheat

Additional file 4: Table S3. Conserved domains of ethylene receptor proteins of hexaploid wheat and its progenitors

Additional file 5: Table S4. Frequency of the occurrence of cis-regulatoryelements in the promoter sequences of ETR genes of hexaploid wheat

## Data Availability

All data generated or analysed during this study are included in this published article and its supplementary information files.

## Abbreviations

ETR: Ethylene receptor protein
GAF: CGMP phosphodiesterases/adenylyl cyclases/FhlA
ROS: Reactive oxygen species
ERS: Ethylene response sensor
CTR1: Constitutive Triple Response
EIN: Ethylene insensitive
ERF: Ethylene response factor
HisKA: His kinase
ADH: Alcohol dehydrogenase
