# Supplementary material for: Genome-wide identification of ethylene receptor protein-coding gene families in wheat and their regulated expression during development and under multiple abiotic stresses
Source: BMC Plant Biol. 2026 Jan 26;26:347. doi: 10.1186/s12870-026-08177-7 (PMC12918298; doi:10.1186/s12870-026-08177-7)
Supplement: Supplementary file 1 — Additional file 1: Fig. S1. Chromosomal location of ETR gene family members of hexaploid wheat. Fig. S2. Evolutionary relationship and exon/intron structural organization of the ETR genes in hexaploid wheat, Arabidopsis and rice. [file 12870_2026_8177_MOESM1_ESM.pdf]

Genome-wide identification of ethylene receptor protein-coding gene families in wheat and their regulated expression during development and under multiple abiotic stresses

Murali Krishna Koramutla[a], Manisha Negi[a], Deepak Sharma[a] and Belay T. Ayele[a*]

[a]Department of Plant Science, 222 Agriculture Building, University of Manitoba, Winnipeg, Manitoba Canada R3T 2N2

Supplementary Figures

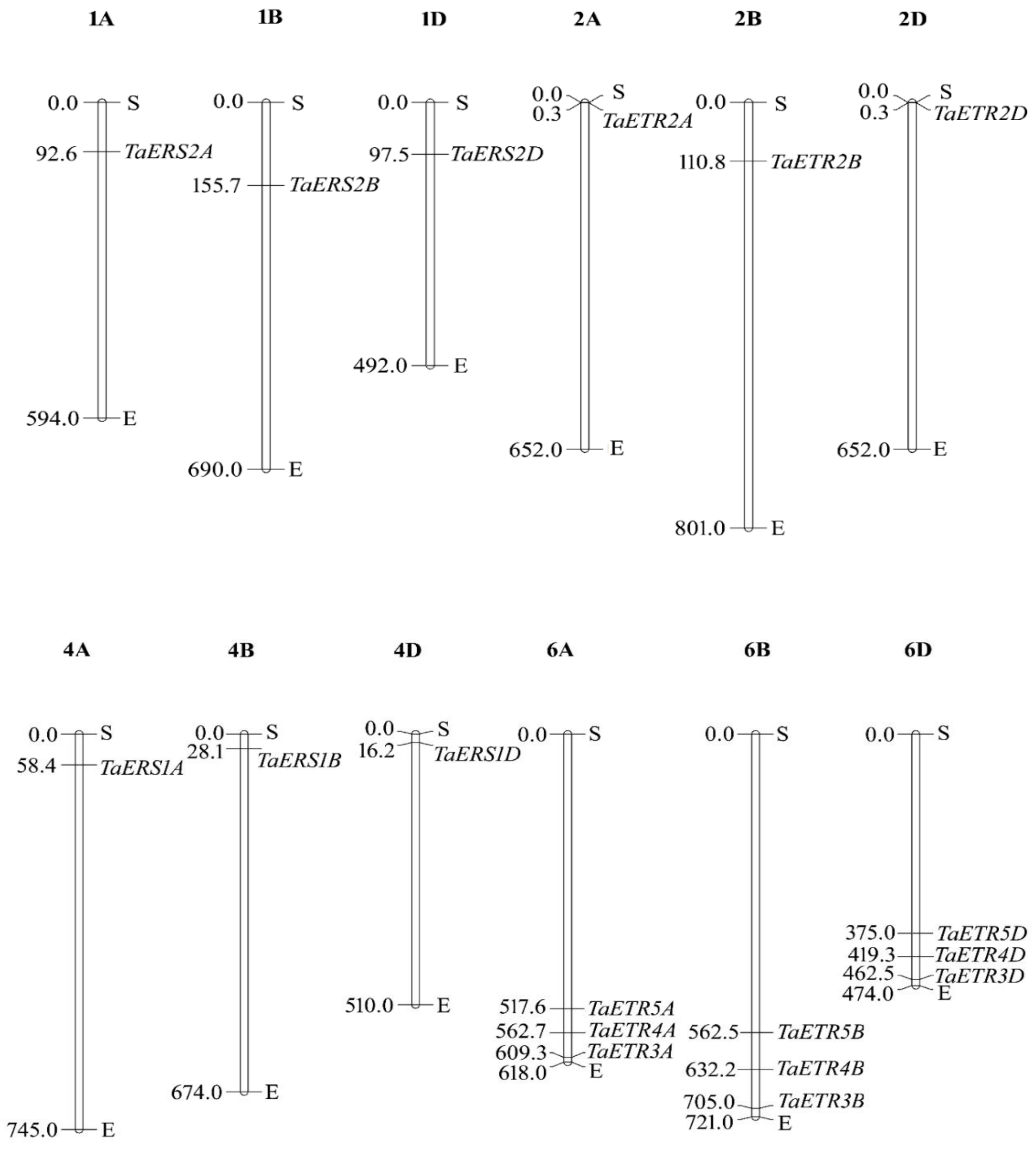

Fig. S1. Chromosomal location of *ETR* gene family members of hexaploid wheat. The chromosomal location of the *TaETR* homoeologs were identified based on the reference genome sequence of bread wheat (IWGSC RefSeq v1.0) available in URGI assemblies database using MapChart 2.32 tool. E at the bottom end of each chromosome represents the size of the chromosome in megabases, and the numbers and letters following the numbers at the top indicate the chromosome number and its subgenome, respectively.

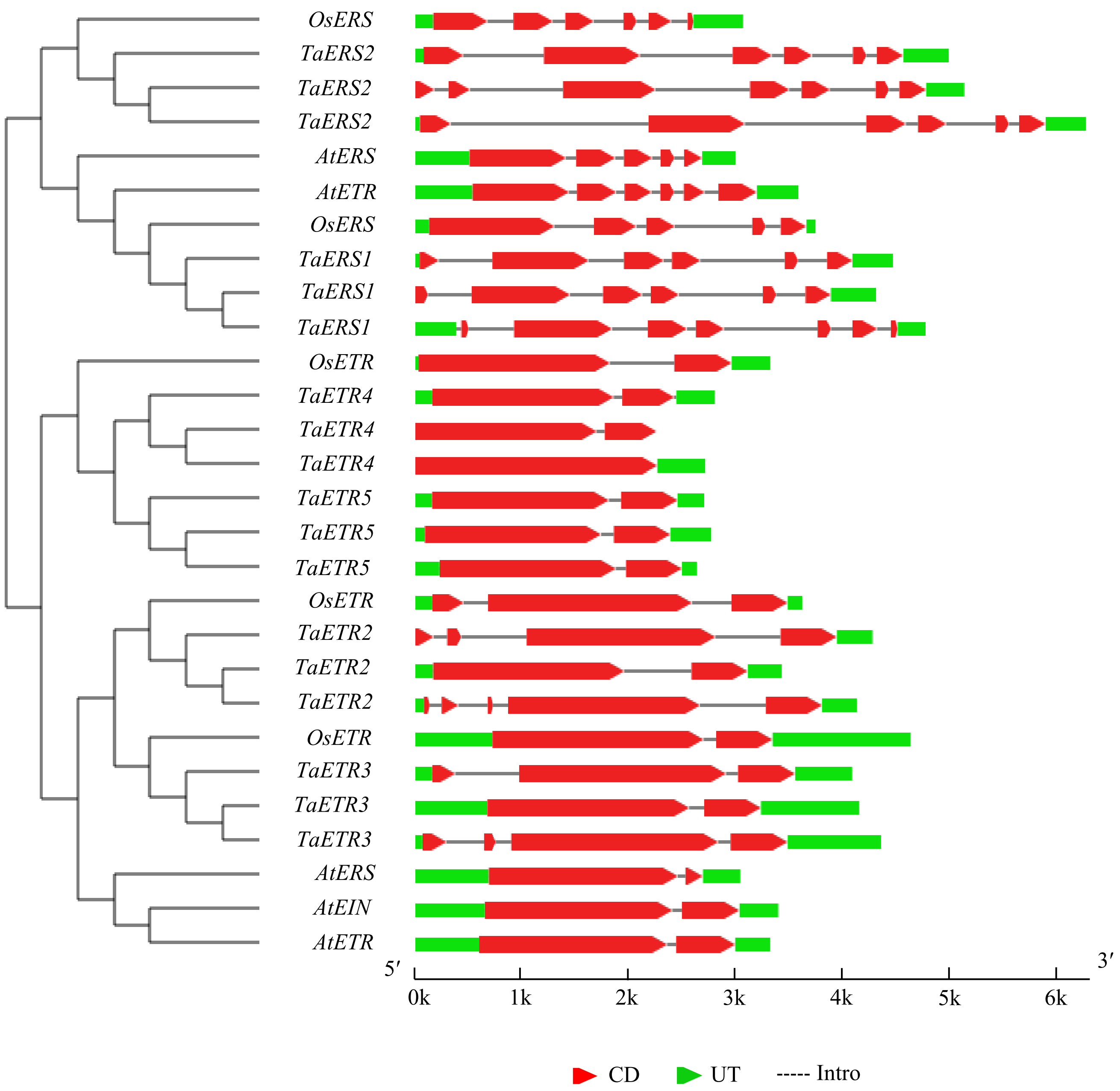

Fig. S2 Evolutionary relationship and exon/intron structural organization of the *ETR* genes in hexaploid wheat, Arabidopsis and rice.
