# Supplementary material for: Genome-wide identification of ethylene receptor protein-coding gene families in wheat and their regulated expression during development and under multiple abiotic stresses
Source: BMC Plant Biol. 2026 Jan 26;26:347. doi: 10.1186/s12870-026-08177-7 (PMC12918298; doi:10.1186/s12870-026-08177-7)
Supplement: Supplementary file 3 — Additional file 3: Table S2. Duplication events of ETR gene family members in hexaploid wheat [file 12870_2026_8177_MOESM3_ESM.pdf]

Table S2 Duplication events of *ETR* gene family members/homoeologs in hexaploid wheat

Gene pairs	Gene Alignment Coverage (%)	Threshold	Identity (%)	Duplication Event
TaERS1A/TaERS1B	100	0	96.96	Segmental
TaERS1A/TaERS1D	100	0	96.86	Segmental
TaERS1B/TaERS1D	100	0	97.06	Segmental
TaERS2A/TaERS2B	100	0	97.29	Segmental
TaERS2A/TaERS2D	100	0	97.5	Segmental
TaERS2B/TaERS2D	99	0	97.54	Segmental
TaETR2A/TaETR2B	100	0	96.45	Segmental
TaETR2A/TaETR2D	100	0	98.33	Segmental
TaETR2B/TaETR2D	100	0	97.08	Segmental
TaETR3A/TaETR3D	100	0	95.67	Segmental
TaETR3A/TaETR3B	100	0	95.54	Segmental
TaETR3B/TaETR3D	100	0	96.18	Segmental
TaETR4A/TaETR4D	100	0	97.45	Segmental
TaETR4A/TaETR4B	100	0	96.58	Segmental
TaETR4B/TaETR4D	100	0	97.21	Segmental
TaETR5A/TaETR5D	100	0	96.25	Segmental
TaETR5A/TaETR5B	99	0	94.47	Segmental
TaETR5B/TaETR5D	100	0	94.47	Segmental
