# Supplementary material for: Genome-wide identification of ethylene receptor protein-coding gene families in wheat and their regulated expression during development and under multiple abiotic stresses
Source: BMC Plant Biol. 2026 Jan 26;26:347. doi: 10.1186/s12870-026-08177-7 (PMC12918298; doi:10.1186/s12870-026-08177-7)
Supplement: Supplementary file 4 — Additional file 4: Table S3. Conserved domains of ethylene receptor proteins of hexaploid wheat and its progenitors [file 12870_2026_8177_MOESM4_ESM.pdf]

Table S3 Conserved domains of ethylene receptor proteins of hexaploid wheat and its progenitors

Species	Receptor Protein	No. of trans membranes	Functional Domains (start to end)				Signal peptide (start to end)
			GAF	HisKA	HATpase_c	REC	
T. aestivum	TaERS1A	3	158-317	343-408	455-589	na	na
	TaERS1B	3	158-317	343-408	455-589	na	na
	TaERS1D	3	158-317	343-408	455-589	na	na
	TaERS2A	3	158-317	343-408	455-589	na	na
	TaERS2B	3	158-317	343-408	455-587	na	na
	TaERS2D	3	158-317	343-408	455-587	na	na
	TaETR2A	2	202-362	388-453	na	655-771	1-36
	TaETR2B	2	193-353	379-444	491-619	646-762	1-27
	TaETR2D	2	200-360	386-451	na	653-769	1-34
	TaETR3A	3	230-369	417-482	529-665	689-802	na
	TaETR3B	3	205-366	392-457	504-640	664-777	1-36
	TaETR3D	3	205-366	392-457	504-640	664-777	1-37
	TaETR4A	3	179-384	374-439	na	611-724	na
	TaETR4B	3	177-346	372-437	na	609-722	na
	TaETR4D	3	177-346	372-437	na	609-722	na
	TaETR5A	3	164-323	na	460-577	599-721	na
	TaETR5B	3	159-318	na	na	600-722	na
	TaETR5D	3	164-323	na	na	597-719	na
T. turgidum	TtERS1A	3	158-317	343-408	455-589	na	na
	TtERS1B	3	158-317	343-408	455-560	na	na
	TtERS2A	3	158-317	343-408	455-589	na	na
	TtERS2B	3	158-317	343-408	455-587	na	na
	TtETR2A	2	202-362	388-453	na	655-771	1-36
	TtETR2B	2	195-355	381-446	na	n/a	1-29
	TtETR3A	3	230-391	417-482	529-665	689-802	na
	TtETR3B	3	205-366	392-457	504-640	664-777	1-36
	TtETR4A	3	179-348	374-439	na	611-724	na
	TtETR4B	3	177-346	372-437	na	609-722	na
	TtETR5A	3	164-323	na	460-577	599-721	na
	TtETR5B	3	159-318	na	na	600-720	na
Ae. tauschii	AeERS1	3	158-317	343-408	455-587	na	na
	AeERS2	3	158-317	343-408	455-587	na	na
	AeETR2	2	201-361	387-452	na	654-770	1-35
	AeETR3	3	205-366	392-457	504-640	664-777	1-37
	AeETR4	3	294-463	489-554	na	726-839	na
	AeETR5	3	164-323	na	na	597-719	na

na, not available
